# Copy number variation analysis in cytochromes and glutathione S-transferases may predict efficacy of tyrosine kinase inhibitors in chronic myeloid leukemia

**DOI:** 10.1371/journal.pone.0182901

**Published:** 2017-09-13

**Authors:** Alexander V. Lavrov, Oksana A. Ustaeva, Elmira P. Adilgereeva, Svetlana A. Smirnikhina, Ekaterina Y. Chelysheva, Oleg A. Shukhov, Yuriy V. Shatokhin, Sergey V. Mordanov, Anna G. Turkina, Sergey I. Kutsev

**Affiliations:** 1 Laboratory of Mutagenesis, Federal State Budgetary Institution Research Centre for Medical Genetics, Moscow, Russian Federation; 2 Department of Molecular and Cellular Genetics, State Budgetary Educational Institution of Higher Professional Education Russian National Research Medical University named after N.I. Pirogov of Ministry of Health of the Russian Federation, Moscow, Russian Federation; 3 Laboratory of Medical Genetics, The Rostov State Medical University, Rostov-on-Don, Russian Federation; 4 Scientific and Advisory Department of Chemotherapy of Myeloproliferative disorders, Federal State-Funded Institution National Research Center for Hematology of the Ministry of Healthcare of the Russian Federation, Moscow, Russian Federation; Universita degli Studi di Firenze, ITALY

## Abstract

Chronic myeloid leukemia (CML) is a myeloproliferative disease characterized by the presence of *BCR/ABL* fusion gene in leukemic cells, which promotes uncontrolled cell proliferation. Up to 20% of CML patients show primary resistance or non-optimal response to tyrosine kinase inhibitor (TKI) therapy. We investigated the association between copy number variation (CNV) in glutathione S-transferases (GST) and cytochromes (CYP) and the response rate to TKI. We enrolled 47 patients with CML: 31 with an optimal response and 16 with failure at 6 months in accordance with European LeukemiaNet 2013 recommendations. CNV detection was performed using SALSA MLPA P128-C1 Cytochrome P450 probe mix. Patients with optimal response and with failure of TKI therapy showed different frequencies of wild type and mutated CYPs and GST (p<0.0013). Validation in the group of 15 patients proved high prognostic value (p = 0.02): positive and negative predictive value 83% and 78%; sensitivity and specificity 71% and 88%. Wild type genotypes of CYP and GST associate with a worse response to TKI treatment in CML patients. This test can be recommended for further clinical trials.

## Introduction

Chronic myeloid leukemia (CML) is one of the most frequent types of leukemia found in 13.7% of newly diagnosed adults [1]. It is characterized by a pathognomonic translocation t(9;22)(q34;q11.2) forming aberrant chromosome 22, the so-called Philadelphia chromosome (Ph+), which carries the *BCR/ABL* fusion gene. BCR/ABL is a constitutively activated tyrosine kinase that drives uncontrolled proliferation, blocks apoptosis and induces genome instability. The incidence rate of CML is approximately 0.7–1.75 cases per 100,000 of population [2]; the raw incidence rate in Europe is 0.99/100,000 [3]. Targeted tyrosine kinase inhibitors (TKI) provide deep and prolonged molecular remission in most CML patients, but many researchers report that primary resistance or non-optimal response to therapy is registered in 5–20% of patients [4, 5].

The causes of primary resistance and slow response have been investigated in numerous studies. There were studied associations of therapy efficacy with expression levels of individual genes [6–11], gene networks at whole transcriptome level [12] and miRNAs [13]. Genetic polymorphisms [14] and whole exomes [15, 16] were also analyzed to find possible prognostic markers. However, so far the results are inconsistent with each other and cannot be used to create a clinically relevant test with additional prognostic markers of treatment response. Studies of metabolizing enzymes reveal some valuable associations between polymorphisms in glutathione S-transferases (GST) and cytochromes (CYP) and the response rate to TKI therapy [17, 18].

Unlike SNP frequently used in association studies, copy number variations (CNV) are currently underresearched although their impact on the gene function could be even more significant due to their physical size.

The aim of our study was to explore the interrelation between CNV in GST and CYP genes and response rate to TKI. We have discovered that CNV in GST and CYP have different frequencies in patients with optimal response and those non-responsive to TKI therapy. CNV analysis of these genes may become a valuable prediction marker of TKI efficiency in CML patients.

## Materials and methods

This study was approved by the Ethical Committee of the Federal State Budgetary Institution “Research Centre for Medical Genetics” of Russian Academy of Medical Sciences (recently renamed Federal State Budgetary Institution “Research Centre for Medical Genetics”). Protocol #6 from July 2nd, 2012.

### Patients and estimating TKI therapy efficacy

We enrolled 47 patients with CML: 31 patients with optimal response and 16 with therapy failure. The response and failure criteria were formed in accordance with ELN2013 recommendations [19]: *BCR-ABL*^IS^<1% and/or Ph+ 0% for optimal response and *BCR-ABL*^IS^>10% and/or Ph+ >35% at 6 months of TKI therapy for the failures. The patients were recruited from the National Research Center for Hematology in Moscow and from Rostov State Medical University, Russia.

Forty-five patients had chronic phase CML, and two were in accelerated phase (both in the failure group). The median age of the patients was 51 years (range 26–70) in the failure group and 39 years (range 19–75) in responders. The sex ratio was 1.7:1 males/females in the failure group and 1.1:1 in responders. Most of the patients (43 out of 47) received imatinib; however, one of the responders started therapy with dasatinib and three more started with nilotinib. Fourteen patients in the failure group and four patients in the responders group received hydroxyurea prior TKI therapy. The general characteristics of the patients are shown in Table 1; more details are available (available upon request because it contains information that could identify study participants).

**Table 1:** Patient profiles.

	Optimal response, n = 31	Non-optimal response, n = 16
Age, years, median (range)	39 (19–75)	51 (26–70)
Male, n (%)	16 (51.6)	10 (62.5)
Female, n (%)	15 (48.4)	6 (37.5)
CML phase		
Chronic phase, n (%)	31 (100)	14 (87.5)
Accelerated phase, n (%)	0 (0)	2 (12.5)
Therapy		
Imatinib, n (%)	27 (87)	16 (100)
Nilotonib, n (%)	3 (10)	0 (0)
Dasatinib, n (%)	1 (3)	0 (0)
Prior therapy with Hydroxyurea, n (%)	4 (12.9)	14 (87.5)

CML = chronic myeloid leukemia

Peripheral blood samples were collected at diagnosis before starting targeted therapy. The patients signed informed written consent forms titled *Investigation of Molecular Reasons for Primary Resistance of CML Patients to TKI Therapy*. The study was conducted in accordance with the Declaration of Helsinki provisions. The control group of blood samples was collected from 53 healthy donors.

### Patients and validating the test

We studied a group of 15 patients with chronic phase CML. Their median age was 48 years (range 19–71); the sex ratio was 2:1 females/males. All patients received imatinib as first line therapy. Thirteen patients received hydroxyurea and one received busulfan prior to TKI (detailed patient characteristics are available upon request because it contains information that could identify study participants). The patients were recruited from Rostov State Medical University. We used the same criteria to estimate TKI therapy efficacy and collected peripheral blood samples at diagnosis after having the patients sign an informed consent form. These patients were not included in the primary study because 9 out of 15 were classified as warning group at 6 months of TKI in accordance with ELN criteria [19], and 6 patients lacked the information necessary for ELN2013 classification at 6 months.

### CYP and GST analysis

CNV detection was performed using SALSA MLPA P128-C1 Cytochrome P450 probe mix (#P128-100R, MRC-Holland, Amsterdam, Netherlands) following the manufacturer’s protocol. A total of 40 exons in 14 genes were checked for CNV presence (Table 2). All genotypes for each exon in every sample are available in S1 Table. From 2 to 4 exons were tested in each gene. In some cases, all exons of the analyzed gene were deleted; in other cases, only one was deleted.

**Table 2:** Analyzed genes.

Gene	Analyzed exons			
*CYP1A1*	exon 1	exon 2	exon 3	
*CYP1A2*	exon 2	exon 4	exon 7	
*CYP1B1*	exon 1	exon 3		
*CYP2A6*	exon 1	exon 2	exon 3	exon 5
*CYP2B6*	exon 2	exon 3	exon 4	
*CYP2C19*	exon 2	exon 6	exon 9	
*CYP2C9*	exon 1	exon 7	exon 8	exon 9
*CYP2D6*	exon 1	exon 5	exon 6	
*CYP2E1*	exon 5	exon 6	exon 8	
*CYP3A4*	exon 1	exon 6	exon 13	
*CYP3A5*	exon 2	exon 4	exon 10	
*GSTM1*	exon 3	exon 5		
*GSTP1*	exon 3	exon 4		
*GSTT1*	exon 1	exon 5		

This can be explained by partial gene CNVs as well as by rare mutations disrupting MLPA-probe binding site. Based on this data CNV status was assigned to the whole gene following this rule: if deletion of either of the exons of the gene was detected, the whole gene was marked as deleted; if duplication of either of the exons was detected, the whole gene was marked as duplicated.

### Statistical analysis

Chi-square criteria was used to determine sensitivity, specificity, positive and negative predictive values for the obtained pattern of genetic markers.

## Results

Three genes (*CYP1A1*, *CYP1B1*, and *CYP2B6*) had no CNV in analyzed exons and were excluded from further analysis. Finally, we analyzed genotypes of 11 genes: *CYP1A2*, *CYP2A6*, *CYP2C19*, *CYP2C9*, *CYP2D6*, *CYP2E1*, *CYP3A4*, *CYP3A5*, *GSTM1*, *GSTP1 and GSTT1* (S2 Table).

All samples were split into 7 categories based on the mutation status of all 11 genes:

1) *wild type* with two copies of each gene;

2) *homozygous deletion* if only deletions in homozygous form were found in one or more of the genes;

3) *heterozygous deletion* if only deletions in heterozygous form were found in one or more of the genes;

4–7) combinations of deletions and duplications in different genes (Table 3).

**Table 3:** Difference in CNV status of CYP and GST genes (*P*< 0.0013) in optimal response, TKI failure and control groups of CML patients.

	Control (n = 53)	Failure (n = 16)	Responder (n = 31)
Wild type	9%	50%	3%
Homozygous deletions	28%	44%	19%
Heterozygous deletions	19%	0%	19%
Hetero- and homozygous deletions	34%	6%	42%
Duplications	2%	0%	0%
Heterozygous deletions and duplications	6%	0%	3%
Hetero- and homozygous deletions and duplications	2%	0%	13%

CML = chronic myeloid leukemia; TKI = tyrosine kinase inhibitor; CNV = copy number variation; CYP = cytochrome; GST = glutathione S-transferase

Three groups of patients (Control Group, CML Failures and CML Responders) differed in frequencies of these categories (*P*<0.0013). The Control Group didn’t differ from the Responders (*P* = 0.41). The most pronounced difference between the failures and the responders was in the frequency of wild type genotype. This observation allowed us to reduce the number of categories to only two: wild type and mutated (deletion, duplication or their combination). We found that in this configuration the test has 50% sensitivity and 97% specificity with positive predictive value (PPV) of 89% and negative predictive value (NPV) of 79% (Table 4).

**Table 4:** Diagnostic value of CNV in *CYP1A2*, *CYP2A6*, *CYP2C19*, *CYP2C9*, *CYP2D6*, *CYP2E1*, *CYP3A4*, *CYP3A5*, *GSTM1*, *GSTP1 and GSTT1* for prediction of optimal response and failure of TKI therapy in CML patients (*P* = 0.0001).

	Failure (n = 16)	Responder (n = 31)	
wild type	8	1	PPV = 89%
non-wild type	8	30	NPV = 79%
	Sensitivity = 50%	Specificity = 97%	

CNV = copy number variation; TKI = tyrosine kinase inhibitor; CML = chronic myeloid leukemia; PPV = positive predictive value; NPV = negative predictive value

We mentioned earlier that CNV in *GSTM1* are frequent in all groups and may be non-specific for prognosing therapy outcome. We excluded *GSTM1* data and, indeed, the difference between Failures and Responders became even more significant (*P* = 6.2*10^−7^ vs *P* = 10^−4^) (Table 5). It is also important that, while slightly reducing the specificity (from 97% to 87%), exclusion of *GSTM1* increased sensitivity from 50% to 88%.

**Table 5:** Diagnostic value of CNV in *CYP1A2*, *CYP2A6*, *CYP2C19*, *CYP2C9*, *CYP2D6*, *CYP2E1*, *CYP3A4*, *CYP3A5*, *GSTP1* and *GSTT1* for prediction of optimal response and failure of standard TKI therapy in CML patients (*P* = 6.2*10^−7^).

	Failure (n = 16)	Responder (n = 31)	
wild type	14	4	PPV = 78%
non-wild type	2	27	NPV = 93%
	Sensitivity = 88%	Specificity = 87%	

CNV = copy number variation; TKI = tyrosine kinase inhibitor; CML = chronic myeloid leukemia;

PPV = positive predictive value; NPV = negative predictive value

### Validation of the CYP-based test in additional group of CML patients

**Fig 1:**
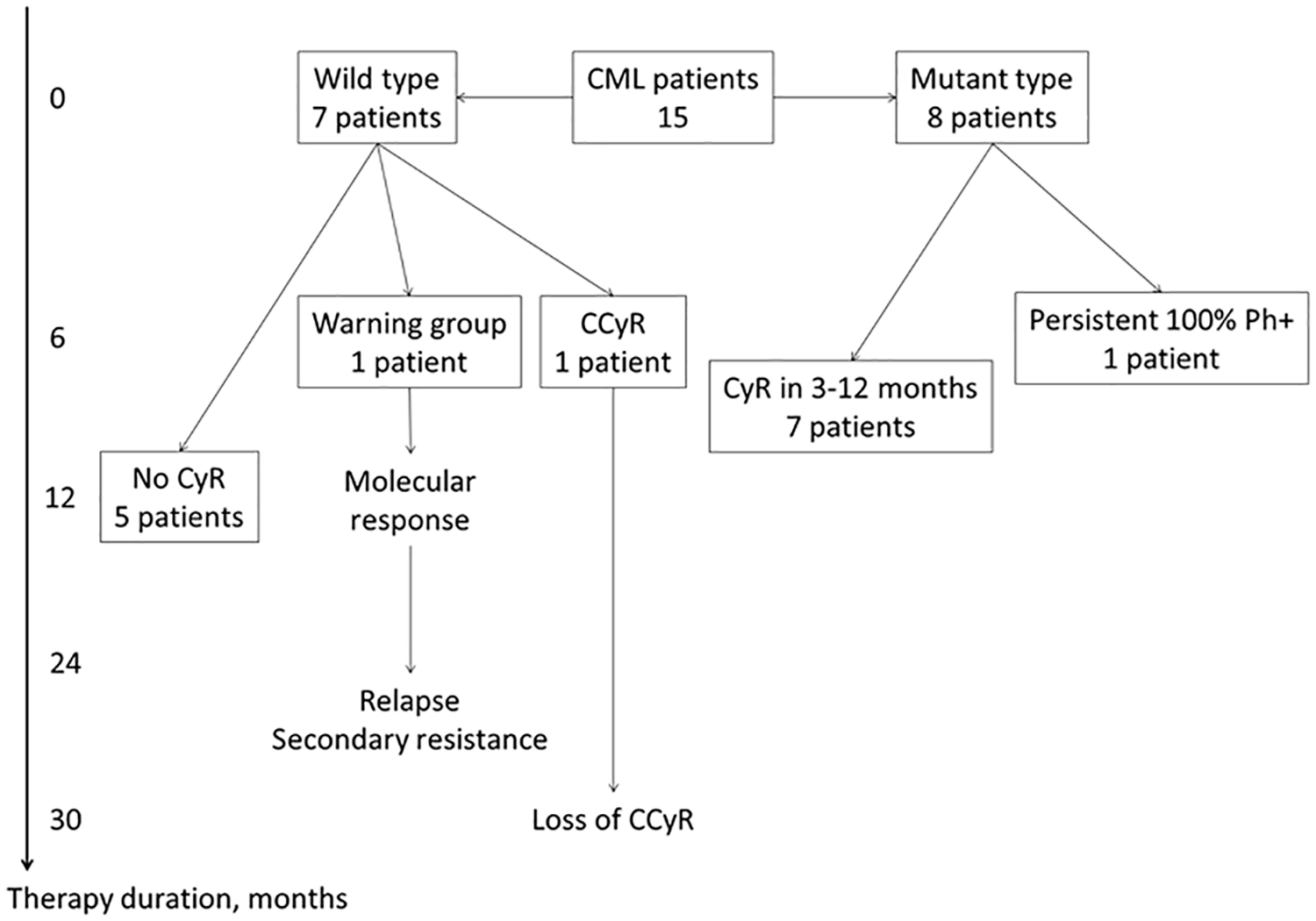
Outcomes of TKIs treatment in validation group of CML patients. CML = chronic myeloid leukemia; CNV = copy number variation; TKI = tyrosine kinase inhibitor; CyR = cytogenetic response; CCyR = complete cytogenetic response.

We used additional patients to validate CNV prognostic testing. We performed CNV analysis and classified these patients as CNV Failures or CNV Optimal Responders depending on their CNV genotypes (Fig 1).

Among 15 CML patients 7 were classified as wild type CNV Failures and 8 as CNV-mutants–CNV Optimal Responders. Five patients from CNV failures did not reach cytogenetic response at 12 months. One patient was in the warning group at 6 months and reached deep molecular response at 12 months. Later, at 24 months, this patient relapsed due to secondary resistance, but he still can be considered a primary responder during the first year of TKI therapy. Another patient had complete cytogenetic response at 6 months and lost it in 2.5 years. There is no data on molecular response but he can also be considered a primary responder at 6 months.

Seven out of 8 CNV Optimal Responders reached complete cytogenetic remission within 3 to 12 months of TKI therapy, and one patient had a persistent 100% Ph+ result of cytogenetic analysis. Available clinical data is summarized in S2 Table.

The outcomes in CNV Failures and CNV Optimal Responders groups were different (*P* = 0.02), and the test demonstrated good characteristics in this small validation dataset: PPV = 83%; NPV = 78%; Sensitivity = 71%; Specificity = 88%.

It should be noted that currently clinical data in this validation is incomplete, and therefore the validation results should be used with caution.

## Discussion

Although CYP3A4 and CYP3A5 play a role in imatinib metabolism, there is currently no reliable data confirming that any variation in these genes may predict therapy outcome in CML [4]. In our study, *CYP3A5* had a homozygous deletion of exon 4 in only one control sample. However, we found CNV mutations in other CYP among optimal responders: *CYP2A6*, *CYP2D6* and *CYP2E1*. The last two enzymes are thought to be frequently involved in drug metabolism [20].

CYP2E1 plays a minor role in imatinib metabolism and can be inhibited by imatinib as well as CYP2D6 [21, 22]. CYP2D6 is widely used for classification of people in poor/rapid metabolizers and demonstrates the largest phenotypic variability among the CYP [23]. CYP2A6 is involved in nicotine metabolism and many drugs; however, there is no data about its interactions with imatinib [24].

Genetic polymorphisms in *CYP3A4* and *CYP3A5* have been studied in 189 CML patients and no association was found with TKI therapy outcomes at 12 months after starting therapy [14]. B. Mitchell and M. Deininger pointed out SNPs in *CYP3A5* among other molecular markers as possible predictors of primary TKI resistance [25]. Several reviewers highlighted the problem of primary resistance in CML patients to TKIs, especially to imatinib as the most long-used and well-studied TKI. D. Bixby and M. Talpaz argued that some inconsistency exists between different studies, and imatinib dose escalation may be helpful under some circumstances [4].

The main difference between our groups of patients is contributed by the CNVs in *GSTT1* (S3 Table). It was previously shown that polymorphisms of the GSTs correlate with a risk of acute myeloid leukemia (AML) in accordance with the meta-analysis of case-control studies published between 1998 and 2009 [26]. Specifically, deletions of *GSTM1* and *GSTT1* were associated with poor prognosis of AML and worse chemotherapy efficacy [27]. Besides genetic polymorphisms of *CYP1A1*, *CYP2D6*, *GSTM1* and *GSTT1* associate with acute lymphoblastic leukemia in Indian children [28].

A systematic review with meta-analysis was performed to investigate the association between GST polymorphisms (*GSTM1*, *GSTP1* and *GSTT1*) and the development of acute leukemia, since the overall results of existing published studies remained inconsistent with each other [29]. The pooled OR of acute leukemia risks associated with *GSTM1* null genotype, *GSTP1* Val105 allele and *GSTT1* null genotype, were 1.22 (95% CI 1.07–1.38), 1.07 (95% CI 1.00–1.13) and 1.19 (95% CI 1.00–1.41), respectively. A significant rise in acute lymphoblastic leukemia risk was observed in patients with *GSTM1* and *GSTT1* null genotypes. The authors suggested that *GSTM1* and *GSTT1*, but not *GSTP1* polymorphisms, were associated with a modest increase in the risk of acute lymphoblastic leukemia and *GSTM1*/*GSTT1* null genotypes could play a role in leukemogenesis [29].

However, more evidence of an association between GST genotypes and AML was revealed during a pooled analysis of 29 studies, suggesting that the *GSTM1*-null genotype associates with an increased risk of AML in East Asians (p = 0.01; OR = 1.22; 95% CI 1.05–1.42), and *GSTT1*-null genotype in Caucasians (p<0.0001; OR = 1.48; 95% CI 1.29–1.69). In addition, the presence of double-null genotypes increased the risk of AML in both Caucasians and East Asians. The authors concluded that inheritable GST status could influence the risk of developing AML [30].

It was also revealed that gain of *GSTT1* copy was more frequent in CML patients who needed imatinib dose escalation because of an initial lack of response to the standard dose [31]. This is consistent with our results where wild type genotypes associate with failure while deletions are typical for optimal responders.

Later it was observed that deletions of *GSTT1* associate with imatinib failure, while CNV in *GSTM1* or *GSTP1* have no influence on risk of failure [32]. The authors of that study estimated failure of treatment during at least 36 months and moreover analyzed patients with secondary failure including confirmed mutations of *BCR/ABL*. It is necessary to analyze primary and secondary resistance separately, since the causes and clinical consequences of these resistances are different. That is why it is complicated to compare the above mentioned results of *GSTT1* CNV analysis with our results where we selected only primary resistance/inefficiency of TKI therapy.

One of the latest studies revealed that *GSTT1/GSTM1* null genotypes are associated with higher rate of cytogenetic response [33]. However, the authors used only one PCR reaction per gene, and only homozygous deletion of either of the genes was detected. This reduces the accuracy of the research.

Our data supports our own clinical experience with early switch of therapy in cases of non-optimal response at 3 months in accordance with ELN2013 criteria. We show that increasing the imatinib dose to 600 mg or switching to other TKIs helps to achieve cytogenetic and molecular remission within 9 to 12 months from starting initial therapy in most of the CML patients [34–35]. Wild type genotypes of the metabolizing genes may provide quick metabolism of TKIs and explain the need for increasing the dose in patients with efficient metabolism of xenobiotics, including imatinib and its derivatives [23, 33].

In this study we suggested that a simple categorization into wild and mutated types of GST and CYP genes can be valuable for prognostic purposes and robust patient classification in high-risk failures and responders. The configuration of the test should be further assessed both with *GSTM1* data (GSTM+) and without *GSTM1* data (GSTM-).

The first configuration demonstrated high PPV (89%) in our sample; however, it should be recalculated reveal the actual prevalence of primary failures among all CML patients [36]. If we assume that primary failures account for 15% of all CML patients, then the corrected PPV of the test in real life will be 74.6% and NPV will be 91.6%. The GSTM test gives corrected PPV of 54.4% and NPV gives 97.6%. These results demonstrate that the test can help filter out patients responding to standard therapy and to focus on those at high risk (54.4% or even 74.6%) of primary non-optimal response and who may need an early change of therapy to either higher doses of TKIs or next-generation TKIs. Selecting patients with good prognosis on standard therapy is also important because this may help avoid unnecessary expenses and keep second and third generations of TKI available for the treatment of possible recurrence of the disease.

In conclusion, wild type genotypes of CYP and GST associate with a worse response in CML patients to TKI treatment. CNV mutations in *GSTT1*, *GSTM1*, *CYP2A6*, *CYP2D6* and *CYP2E1* are typical for patients with optimal response to TKI therapy. Testing CNV genotypes of these genes demonstrates excellent PPV (83%) and NPV (78%) in predicting response to TKI therapy in CML and can be recommended for further investigation in clinical trials.

We anticipate that testing for CNVs in GSTs and CYPs can become the tool to select patients for different starting dose regimen.

## Supporting information

S1 Table CYP and GST genotypes. (XLSX)

S2 Table 11 genes genotype analysis (CYP1A2, CYP2A6, CYP2C19, CYP2C9, CYP2D6, CYP2E1, CYP3A4, CYP3A5, GSTM1, GSTP1, and GSTT1). (XLSX)

S3 Table Differences between groups of patients due to CNVs in GSTT1. (XLSX)
